# Association between Early Absolute Neutrophil Count and Level of D-Dimer among Patients with COVID-19 Infection in Central Taiwan

**DOI:** 10.3390/jcm10173891

**Published:** 2021-08-30

**Authors:** Wen-Cheng Chao, Chieh-Liang Wu, Jin-An Huang, Jyh-Wen Chai, Chieh-Lin Teng, Wen-Lieng Lee, Yun-Ching Fu, Shih-Ann Chen

**Affiliations:** 1 Department of Critical Care Medicine, Taichung Veterans General Hospital, Taichung 40705, Taiwan; cwc081@hotmail.com (W.-C.C.); cljeff.wu@gmail.com (C.-L.W.); 2 Department of Computer Science, Tunghai University, Taichung 407224, Taiwan; 3 Department of Automatic Control Engineering, Feng Chia University, Taichung 407802, Taiwan; 4 Big Data Center, National Chung Hsing University, Taichung 40227, Taiwan; 5 Department of Industrial Engineering and Enterprise Information, Tunghai University, Taichung 407224, Taiwan; 6 Department of Neurological Institute, Taichung Veterans General Hospital, Taichung 40705, Taiwan; jahuang@vghtc.gov.tw; 7 Department of Radiology, Taichung Veterans General Hospital, Taichung 40705, Taiwan; hubt@vghtc.gov.tw; 8 Division of Hematology and Medical Oncology, Department of Medicine, Taichung Veterans General Hospital, Taichung 40705, Taiwan; drteng@vghtc.gov.tw; 9 Department of Life Science, Tunghai University, Taichung 407224, Taiwan; 10 School of Medicine, Chung Shan Medical University, Taichung 40201, Taiwan; 11 Cardiovascular Center, Taichung Veterans General Hospital, Taichung 40705, Taiwan; epsachen@ms41.hinet.net; 12 Department of Medicine, National Yang-Ming Chiou Tung University, Taipei 112, Taiwan; 13 Section of Pediatric Cardiology, Department of Pediatrics, Taichung Veterans General Hospital, Taichung 40705, Taiwan; 14 Institute of Clinical Medicine, National Yang Ming Chiou Tung University, Taipei 112, Taiwan

**Keywords:** COVID-19, Asian, thromboembolism, absolute neutrophil count, D-dimer

## Abstract

Thromboembolism is a critical event in patients with coronavirus disease (COVID)-19 infection and highly associated with neutrophil extracellular traps. D-dimer has been found to be an essential thromboembolism-associated biomarker; however, the association between absolute neutrophil count (ANC) and level of D-dimer in patients with COVID-19 infection remains unclear. In this study, we enrolled consecutive patients with COVID-19 admitted to Taichung Veterans General Hospital (TCVGH), a referral center in central Taiwan with 20 airborne infection isolation rooms. Spearman correlation was used to determine the association between ANC and level of D-dimer in distinct time periods. A total of 28 consecutive patients with COVID-19 infection were enrolled, and 32.1% (9/28) of them required mechanical ventilation. Patients requiring mechanical ventilation had a higher ANC (8225 vs. 3427/µL, *p* < 0.01) and levels of D-dimer (6.0 vs. 0.6 mg/L, *p* < 0.01) compared with those without mechanical ventilation. Notably, we identified five patients with image-proven thromboembolic events during the hospital course, with the number of patients with pulmonary embolism, venous thrombosis and acute ischemic stroke were 2, 1, and 2, respectively. We found that ANC within 4 days correlated with the level of D-dimer to a moderate level (r = 0.71, *p* < 0.05), and the association between ANC and D-dimer no longer exist after day 5. In conclusion, we found highly prevalent thromboembolic events among patients with severe COVID-19 infection in central Taiwan and identified the association between early ANC and D-dimer. More studies are warranted to elucidate the underlying mechanism.

## 1. Introduction

Coronavirus disease 2019 (COVID-19) infection leads to a tremendous impact on public health worldwide and has spread throughout Taiwan, where the number of laboratory-confirmed cases increased abruptly from 1132 on 19 May 2021 to 14,804 on 30 June 2021, despite the strenuous efforts to halt the transmission of COVID-19 in Taiwan [1]. There is a wide range of clinical manifestations that result from distinct inflammatory responses in patients with COVID-19 infection [2,3]. Recent evidence has found highly prevalent thromboembolic events, including deep venous thrombosis (DVT), pulmonary embolism (PE) and acute ischemic stroke, in patients with COVID-19 infection, and D-dimer has been attributed as an essential biomarker for thromboembolic events in patients with COVID-19 infection [4,5,6]. Miro et al., investigating 74,814 patients with COVID-19 with 1,388,879 non-COVID-19 controls in Spain, recently reported that the incidence of PE in patients with COVID-19 was approximately nine-fold higher than those in the non-COVID-19 population (310 vs. 35 per 100,000 person-years) [7].

Unlike a consistent finding with an apparently high prevalence of thromboembolic events among patients with COVID-19 infection in western countries, the ethnic difference remains an essential issue with regard to the prevalence of thromboembolic events and the need for routine thromboprophylaxis among patients with COVID-19 infection in Asian countries, highlighting the need for real-world data on thromboembolic events in the Asian population [7,8,9,10]. Additionally, recent studies have implicated neutrophilic inflammatory response, particularly neutrophil extracellular traps (NETs), with the pathogenesis of thrombosis formation in COVID-19 infection [11]. D-dimer is a biomarker in patients with thromboembolism; however, the association between the absolute neutrophil count (ANC) and D-dimer in patients with COVID-19 infection remains underexplored [11,12]. In the present study, we aim to address the association between ANC and level of D-dimer among patients with COVID-19 infection in central Taiwan.

## 2. Materials and Methods

### 2.1. Ethics Approval

This study was approved by the Institutional Review Board of the Taichung Veterans General Hospital (TCVGH: CE21284A). The informed consent was waived by the IRB due to the retrospective study nature.

### 2.2. Subjects and Study Design

This retrospective study was conducted at TCVGH, a tertiary-care referral hospital with 20 airborne infection isolation rooms, in central Taiwan. We enrolled consecutive patients admitted to TCVGH between May 2021 and June 2021 with laboratory-confirmed COVID-19 by Taiwan Center for Disease Control (CDC).

### 2.3. Managements

The management and laboratory measurement for patients with COVID-19 infection was in accordance with the guideline released by Taiwan CDC, including remdesivir for patients requiring oxygen therapy, dexamethasone 6 mg/day for 10 days in patients requiring oxygen, and tocilizumab (8 mg/kg) for those whose serum C-reactive protein (CRP) was higher than 7.5 mg/dL. The aforementioned guideline released by Taiwan CDC does not specify routine thromboprophylaxis due to the lack of solid evidence in Asian populations; therefore, the use of anticoagulant was a decision of the multidisciplinary discussion at TCVGH in the present study. TCVGH established a multidisciplinary COVID-19 care team. In the present study, the need for anticoagulant was based on the aforementioned multidisciplinary discussion. In brief, we closely monitored the level and D-dimer and administered prophylactic anticoagulant with enoxaparin 40 mg/day among patients whose D-dimer was higher than 3.0 ug/mL, approximately 6× the upper limit of normal [13]. In patients with highly elevated D-dimer or signs of a thromboembolic event, the needed examinations, including computed tomography angiography (CTA) and magnetic resonance angiography (MRA), were arranged to precisely diagnose the thromboembolic event.

### 2.4. Sensitivity Analyses

This study aims to explore the association between ANC and level of D-dimer in patients with COVID-19 infection, and the association may vary with the disease course of COVID-19 infection. We hence used distinct time points, including day 1–3, 1–4 and 1–5, to define the early phase of hospitalization for COVID-19 infection and examined the robustness of the association between early ANC and level of D-dimer in distinct definitions.

### 2.5. Statistical Analyses

Data were expressed as frequencies (percentages) for categorical variables and as median (interquartile range, IQR) for continuous variables. Differences between the two groups were analyzed using the Mann–Whitney U test for continuous variables and Fisher’s exact test for categorical variables. The Spearman correlation was used to determine the correlation between ANC, absolute lymphocyte count (ALC) as well as levels of D-dimer, ferritin, CRP, and lactate dehydrogenase (LDH). Statistical analyses were two-sided, and the level of significance was set at 0.05. Data analysis were conducted using R version 3.6.0.

## 3. Results

### 3.1. Patient Characteristics

A total of 28 consecutive patients with COVID-19 infection was enrolled, and 32.1% (9/28) required mechanical ventilation (Table 1). The mean age of enrolled subjects was 60.0 (50.3–69.5) years, and 39.3% (11/21) were male. With regards to the initial laboratory findings, we found that patients requiring mechanical ventilation had a higher neutrophil count (8225 vs. 3427/mL, *p* < 0.01) and levels of D-dimer (6.0 vs. 0.6 mg/L, *p* < 0.01) compared with those without mechanical ventilation. The levels of C-reactive protein (CRP), ferritin and LDH appeared to be similar between the two groups. Collectively, the severity of enrolled patients with COVID-19 infection in the present study was high, and those requiring mechanical ventilation had a higher neutrophil count and level of D-dimer than those without mechanical ventilation.

### 3.2. Management and Outcome in Patients with COVID-19 Infection

The majority (75%, 21/28) of patients received remdesivir and dexamethasone due to the requirement for oxygen support (Table 2). Furthermore, 42.9% of enrolled subjects underwent tocilizumab given that the level of CRP was higher than 7.5 mg/dL. Notably, we identified five patients with image-proven thromboembolic events during the hospital course, with the number of patients with PE, DVT and acute ischemic stroke were 2, 1, and 2, respectively. We summarized the individual image and dynamic of biomarkers of the five patients with the thromboembolic event in the following section. In this study, 42.9% (12/21) underwent prophylactic anticoagulant and 100% (9/9) of patients requiring mechanical ventilation received prophylactic anticoagulant. The safety of anticoagulant tended to be high, only one patient had major bleeding (tarry stool requiring blood transfusion more than 2 units) and two patients had minor bleeding (hematuria and ecchymosis). All the enrolled patients in this study survived, and the overall hospital-day and ventilator-day were 15.5 (13.0–22.8) days and 10.0 (7.5–19.5) days, respectively.

### 3.3. Thromboembolic Events in 5 Patients with COVID-19 Infection

**Figure 1:**
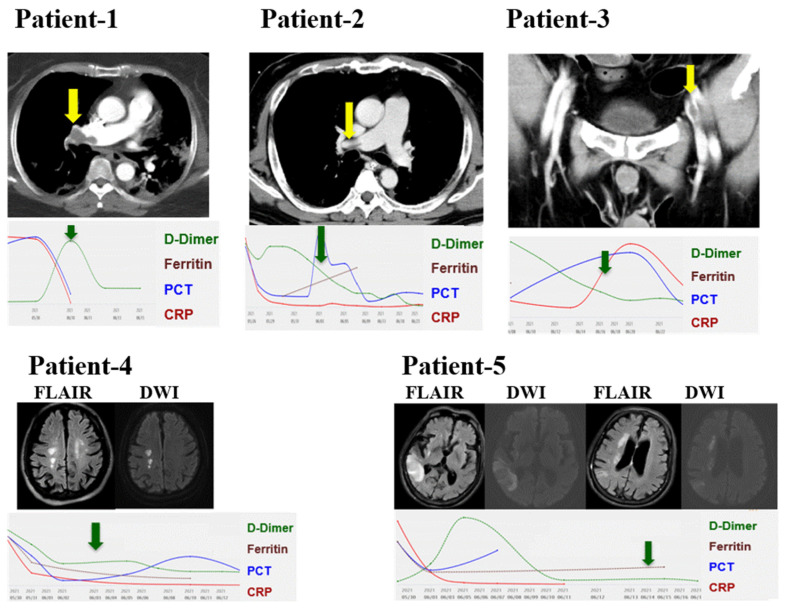
Image and trends of biomarkers among the five patients with thromboembolic events. Pulmonary embolism, patient-1 and patient-2; femoral venous thrombosis, patient-3; acute ischemic stroke, patient-4 and patient-5. Green arrow indicates the date of image survey and yellow arrow represent the presence of thromboembolism. FLAIR, fluid-attenuated inversion recovery; DWI, diffusion-weighted image.

Figure 1 illustrates the image and dynamic of biomarkers for COVID-19 in the five patients with thromboembolic events (Figure 1). With regards to the two patients diagnosed with acute ischemic stroke after COVID-19 infection. Patient-4 was found to have left hemiplegia after the discontinuation of sedation, and MRA was arranged after the stabilization of critical illness. Similarly, patient-5, who received HFNC due to oxygenation failure, complained of left hemiparesis (muscle power 3+) after the improvement of the oxygenation and MRA was arranged to ascertain the etiology of left hemiparesis. Taken together, we found highly prevalent thromboembolic events among patients with severe COVID-19 infection in the present study.

### 3.4. Correlation between ANC and Level of D-Dimer

**Figure 2:**
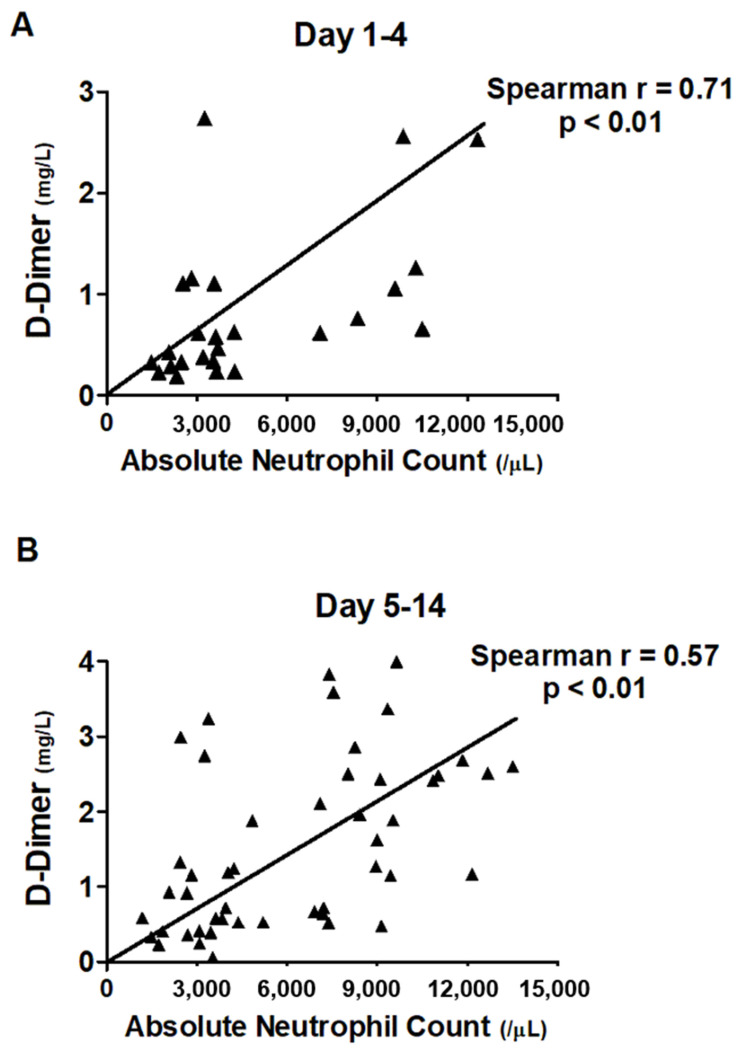
Correlation between the level of D-Dimer and neutrophil count among patients with COVID categorized by hospital day: (**A**) day 1–4, (**B**) day 5–14.

We found an increased ANC and elevated level of D-dimer in COVID-19 requiring mechanical ventilation (Table 1). We hence focused on exploring the association between ANC and level of D-dimer in the distinct disease course. We measured the association among ANC, ALC, and levels of COVID relevant biomarkers, including CRP, ferritin, LDH, and D-dimer (Table 3). We found that ANC tended to be slightly associated with the level of D-dimer, with the Spearman correlation coefficient at 0.58. We then categorized the disease course into acute (day 1–4), sub-acute (day 5–14), and chronic (day 15–28) phases and found that the association between increased ANC and elevated level of D-dimer mainly existed in the early phase of COVID-19, with the Spearman correlation coefficient at 0.71 (Figure 2). In the sensitivity analyses, the moderate association between early ANC and levels of D-dimer appeared to be robust using the distinct definition for the early phase of COVID-19 infection (Supplemental Figure S1).

## 4. Discussion

Thromboembolic event is an emerging and substantial issue in patients with COVID-19 infection, but data in the Asian population remains limited. In the present study, we found that early ANC correlated with the level of D-dimer, whereas ANC after day 5 no longer correlated with the level of D-dimer. This real-world evidence demonstrates the crucial role of active surveillance to diagnose the thromboembolic event and the association between early ANC and level of D-dimer in patients with COVID-19 infection.

The ethnic disparity is currently a concern with regard to the prevalence of the thromboembolic event and the need for prophylactic anticoagulant among patients with COVID-19 in Asian populations [9,10]. Previous studies have shown that venous thromboembolism (VTE), including DVT and PE, seems relatively uncommon in the Asian population [14,15], whereas the bleeding risk among patients receiving anticoagulants tends to be higher in Asians compared with those in Caucasians [16]. However, recent studies have shown that the incidence of VTE in Asian countries, including Taiwan, has risen in the past two decades, and the increased incidence may be attributed to increased vigilance for VTE, ageing population, increased patients with cancer, and altered lifestyle [17,18,19,20]. Notably, accumulating evidence have shown the highly prevalent thromboembolic events in patients including the Asian population with COVID-19 infection, particularly those with high disease severity and elevated levels of D-dimer [4,8,21,22]. Ren et al. carried out a cross-sectional study with a routine survey for DVT using compression ultrasound examinations among 48 patients with severe COVID-19 infection requiring ICU admission in Wuhan [4]. Ren et al. reported that DVT in the lower extremity was detected in 85.4% (41/48) of patients, with 36 having isolated distal DVTs and 5 with proximal DVT, despite all but 1 patient who was contradicted for anticoagulant underwent prophylactic anticoagulant with 30 to 40 mg enoxaparin [4]. Nevertheless, discordant data with regards to the prevalence of VTEs among patients with COVID-19 in Asian countries were found, and such discordant data may result from distinct severity of COVID-19 and intensity of surveillance among studies. Yamashita et al., conducting questionnaire surveillance involving 1243 patients with COVID-19 among 77 institutions in Japan, reported a low prevalence of venous thrombosis (0.6%, 7/1243) and PE (0.4%, 5/1243) [10]. In line with our data that the proportion of thromboembolic events was up to 44.4% (4/9) in patients with severe COVID-19 infection requiring mechanical ventilation, one Japanese multicenter cohort study with 1236 patients with COVID-19 infection found that the prevalence of CT-proven venous thromboembolism was up to 40% in those with severe COVID-19 infection requiring mechanical ventilation [22]. Therefore, more studies for thromboembolic events are warranted to address the epidemiology of thromboembolic events among patients with COVID-19 in Asian populations, particularly studies with active surveillance.

Indeed, it is substantial to address the prevalence of thromboembolic events among patients with COVID-19 in the Asian population, given that physicians in Asia tend to be reluctant to prescribe oral anticoagulants. Oldgren et al., investigating data of 15,400 patients during 2008–2012 with atrial fibrillation in 46 countries, found that the proportion of using oral anticoagulant among patients with atrial fibrillation and CHADS2 score higher than 2 but without a history of rheumatic fever in North American, Western Europe and China were 65.7%, 63.2% and 11.2%, respectively [23]. In the present study, a multidisciplinary COVID-19 care team consisting of the intensivist, cardiologist, neurologist and radiologist was established; therefore, the multidisciplinary team enables us to aggressively diagnose patients with thromboembolic events and to administer optimal both prophylactic and therapeutic anticoagulant as evidence by that all of COVID-19 requiring intubation underwent anticoagulants and merely one patient had major bleeding (Table 1) [24].

The prevalence of VTE in patients with COVID may be affected not only by disease severity but also by the intensity of surveillance. Mumoli et al. recently reported the incident VTE events during the first wave (February 2020 to April 2020, *n* = 316) and second wave (October 2020 to December 2020, *n* = 160) with comparable disease severity at an Italian hospital [13]. They found that the use of CTA to diagnose PE increased from 3.8% (12/316) to 8.8% (14/160), and the application of compression ultrasound to diagnose DVE also increased from 45% (143/316) to 71% (114/160) [13]. Therefore, the increased prevalence of VTE from 13.9% (44/316) to 18.1% (29/160) might at least partly result from increased vigilance and active surveillance for VTE in patients with COVID-19. In the present study, the identification of PE in patient-2 and DVT in patient-3 was based on high vigilance instead of symptomatic VTE, and the thromboembolic event in these two patients might be undetected without timely surveillance (Figure 1). Furthermore, the application of MRA enables us to explicitly diagnose acute ischemic stroke in patients with COVID-19 infection [25]. Collectively, the aforementioned evidence suggests the substantial need for vigilance and timely image surveillance of the thromboembolic event in patients with COVID-19 infection, particularly those with high disease severity.

In line with our study, the increased ANC and elevated neutrophil/lymphocyte ratio has been found to be associated with high disease severity and unfavorable outcomes in patients with COVID-19 infection [26,27,28,29]. Zhang et al. developed a severity prediction model in patients with COVID-19 infection using age and clinical laboratory data, of which white blood cell count and ANC were the two determinants with the highest hazard ratio [26]. In addition to the association between an increased neutrophil count and disease severity of COVID-19 and highly prevalent thromboembolic events in those with severe COVID-19 infection, recent studies have explored underlying neutrophil-associated biological mechanisms, mainly neutrophil extracellular traps (NETs), to link neutrophilic inflammation with thromboembolic events in patients with COVID-19 infection [30,31,32]. The neutrophil is a fundamental immune cell in innate immunity, and NETs, which consist of the release of extracellular chromatin meshes and antimicrobial peptide granules, neutrophil elastase, myeloperoxidase and the other enzymes of neutrophils, play a crucial role in immobilizing microorganisms and orchestrating the activation of adaptive immunity [33,34]. Previous studies in the field of cardiovascular disease have shown the sustained formation of NETs is associated with thromboembolic events through triggering a cascade of inflammatory reactions that lead to the formation of thrombosis [35], and recent evidence has found the crucial role of NETs in the development of thromboembolic events in patients COVID-19 infection through linking dysregulated neutrophilic inflammation and activation of coagulation cascades, so-called immunothrombosis [11]. In the present study, the positive association between ANC and D-dimer merely existed in the early phase among patients with COVID-19 infection, and we postulate that the lack of association between ANC and D-dime after the acute phase might possibly result from the administration of dexamethasone and tocilizumab given that the majority of enrolled subjects in the study hospital were those with severe COVID-19 infection. These findings provide clinical evidence with respect to the increased awareness of NET-associated hypercoagulation in patients with COVID-19 infection [27,30].

Unlike the extensive evidence to report the prevalence of PE and DVT in patients with COVID-19 infection [5,36], the prevalence of stroke in patients with COVID-19 infection is less likely to be addressed [6,37,38]. The reported prevalence of ischemic stroke varied with studies and ranged from 1.3% to 4.6% [6,38]. Li et al. conducted a single center study in Wuhan with 219 patients with COVID-19 infection between January 2020 and February 2020 and reported that 4.6% (10/219) had acute ischemic stroke; the number of patients diagnosed as large vessel occlusion, small vessel occlusion and cardioembolic type were 5, 3 and 3, respectively [6]. Similar to our data, those with acute stroke had a higher level of D-dimer than those without acute stroke (6.9 vs. 0.5 mg/L, *p* < 0.001) and were more likely to have DM (54.5% vs. 12.0%, *p* < 0.01) and hypertension (81.8% vs. 22.1%, *p* < 0.01) [6]. In contrast to the high prevalence of ischemic stroke in the study conducted by Li et al., Qureshi et al. used a claim database involving 8163 patients with confirmed COVID-19 infection and found the prevalence of diagnosis with acute ischemic stroke was 1.3% (103/8163), but using diagnosis code might potentially underestimate the true prevalence of acute ischemic stroke, particularly those with non-severe symptoms [38]. Similarly, Qureshi et al. also found that those with acute ischemic stroke were more likely to have diabetes mellitus (56.3% vs. 30.2%, *p* < 0.01) and hypertension (84.5% vs. 48.2%, *p* < 0.01) compared with those without stroke [38]. Given that the study hospital is a referral center and the enrolled subjects tended to have comorbidities and high disease severity, the two patients with acute ischemic stroke in the present study both have diabetes mellitus, hypertension as well as high disease severity, with one patient receiving intubation and prone ventilation and the other undergoing HFNC. These data indicate the need for vigilance of early detection of stroke among COVID-19 patients, particularly those with multiple comorbidities and high disease severity.

There are limitations in this study. First, the data of this single center study may not be generalizable to the population other than central Taiwan. However, the data analyzed in this study are real-world data obtained in routine care for patients with COVID-19 infection, and the issue of generalization should be at least partly mitigated. Second, due to the observational nature of this study, we were unable to make causal inferences with regard to increased ANC and the elevated level of D-dimer. More large-scale studies, particularly NETs relevant studies, are warranted to validate our finding and to elucidate the underlying biological mechanisms. Third, the lack of protocolized anticoagulants given that the indication, medication, and dosage of thromboprophylaxis among COVID-19 infection remains an inconclusive issue in Asian populations. Fourth, there was a lack of biomarkers, such as Interleukin-6, in this real world study.

## 5. Conclusions

The high vigilance and timely surveillance for the thromboembolic event is an essential issue in the management of patients with COVID-19 infection. In the present study, we found highly prevalent thromboembolic events in patients with severe COVID-19 infection and identified that the ANC within 4 days was correlated with the level of D-dimer. These findings indicate the inclusion of ANC/D-dimer as risk stratification for thromboembolic events in patients with COVID-19 infection, particularly those with high disease activity.

## Figures and Tables

**Table 1:** Characteristics of 28 patients with COVID-19 infection between May 2021 and June 2021 at TCVGH categorized by the requirement of mechanical ventilation.

	All	Mechanical Ventilation (+)	Mechanical Ventilation (−)	*p*-Value
	*n* = 28	*n* = 9	*n* = 19	
COVID severity				
Mechanical ventilation	9	9		
High flow nasal cannula	4		4	
Venturi mask	1		1	
Nasal cannula	7		7	
Room air, lung infiltration	2		2	
Room air, no pulmonary infiltration	5		5	
Prone ventilation				
Awake prone	6		6	
Intubated prone ventilation	2	2		
ARDS grade				
Mild	3	3 (33.3%)	NA	
Mod	3	3 (33.3%)	NA	
Severe	3	3 (33.3%)	NA	
Basic data				
Age, years	60.0 (50.3–69.5)	62.0 (59.5–73.5)	58.0 (40.0–67.0)	0.05
Sex, male	11 (39.3%)	5 (55.6%)	6 (31.6%)	0.41
Body weight, kg	62.5 (59.0–71.8)	70.0 (66.0–77.5)	60.0 (57.0–71.0)	0.04
Symptom-admit, day	5.0 (2.0–8.0)	7.0 (6.0–8.5)	4.5 ± 1.0	0.03
Symptom-intubation, day	7 (3.0–7.0)	7.0 (3.0–7.0)	NA	
Comorbidities				
Diabetes mellitus	8 (28.6%)	4 (44.4%)	4 (21.1%)	0.20
Hypertension	8 (28.6%)	3 (33.3%)	5 (26.3%)	0.52
Coronary artery disease	2 (7.1%)	1 (11.1%)	1 (5.3%)	0.55
Old cerebrovascular diseases	2 (7.1%)	1 (11.1%)	1 (5.3%)	0.55
History of cancer	1 (3.6%)	0 (0%)	1 (5.3%)	0.68
Hepatitis	2 (7.1%)	1 (11.1%)	1 (5.3%)	0.55
COPD	2 (7.1%)	2 (22.2%)	0 (0%)	0.10
Autoimmune disease	1 (3.6%)	0 (0%)	1 (5.3%)	0.70
Laboratory data				
White blood cell counts (/μL)	5430 (4040–8902)	9480 (5300–12,070)	4540 (3950–7200)	0.02
Absolute neutrophil counts (/μL)	3678 (2652–7210)	8225 (4146–12,491)	3427 (2329–4255)	<0.01
Absolute lymphocyte counts (/μL)	913 (604–1260)	843 (312–1232)	958 (618–1341)	0.47
Hemoglobin (g/dL)	13.1 (12.2–14.3)	12.3 (10.6–13.2)	13.3 (12.6–14.4)	0.04
Platelet (10^3^/μL)	201 (156–251)	230 (159–300)	200 (155–234)	0.16
C-reactive protein (mg/dl)	4.4 (12.5–9.39)	9.6 (1.3–15.8)	3.0 (1.1–6.7)	0.12
Ferritin (ng/mL)	875 (162–1328)	1023 (437–1341)	504 (111–504)	0.24
D-Dimer (mg/L)	1.0 (0.4–5.8)	6.0 (4.4–24.6)	0.6 (0.3–1.2)	<0.01
LDH (U/L)	426 (281–570)	533 (496–613)	370 (245–522)	0.02
Procalcitonin (ng/mL)	0.07 (0.05–0.20)	0.12(0.06–0.35)	0.06 (0.05–0.14)	0.19
Creatinine (mg/dL)	0.7 (0.7–0.9)	0.8 (0.7–1.5)	0.7 (0.7–0.8)	0.6

Data were presented as median (interquartile range) and frequency (percentage). Abbreviations: COVID, Coronavirus disease 2019; TCVGH, Taichung Veterans General Hospital; ARDS, acute respiratory distress syndrome; COPD, chronic obstructive pulmonary disease; LDH, lactate dehydrogenase.

**Table 2:** Management and outcome of enrolled patients with COVID infection categorized by the need for mechanical ventilation.

	All	Mechanical Ventilation (+)	Mechanical Ventilation (−)	*p*-Value
	*n* = 28	*n* = 9	*n* = 19	
Management				
Remdesivir, 5-day course	21 (75%)	9 (100%)	12 (63.2%)	0.06
Dexamethasone, 6 mg/day 10 days	21 (75%)	9 (100%)	12 (63.2%)	0.06
Tocilizumab, 8 mg per kg	12 (42.9%)	9 (100%)	3 (15.8%)	<0.01
Vasopressor	6 (21.4%)	6 (66.7%)	0 (0%)	<0.01
Vasopressor-day	3.0 (2.5–4.3)	3.0 (2.5–4.3)		
Sedation	6 (21.4%)	6 (66.7%)	0 (0%)	<0.01
Sedation-day	8.0 (5.8–12.0)	8.0 (5.8–12.0)		
NMBA	6 (21.4%)	6 (66.7%)	0 (0%)	<0.01
NMBA-day	1.0 (0.0–3.5)	1.0 (0.0–3.5)		
Coagulation-associated variables				
Thromembolic events	5 (17.9%)	4 (44.4%)	1 (5.3%)	0.04
Pulmonary embolism	2 (7.1%)	2 (22.2%)	0 (0%)	
Ischemic stroke	2 (7.1%)	1 (11.1%	1 (5.3%)	
Venous thrombosis	1 (3.6%)	1 (11.1%)	0 (0%)	
Anticoagulant	12 (42.9%)	9 (100%)	3 (15.8%)	<0.01
Bleeding events				0.03
Major bleeding	1 (3.6%)	1 (11.1%)	0 (0%)	
Minor bleeding	2 (7.1%)	2 (22.2%)	0 (0%)	
Infection-associated events				
Co-infection	0 (0%)	0 (0%)	0 (0%)	>0.99
Secondary infection	3 (10.7%)	3 (33.3%)	0 (0%)	0.03
Blood stream infection	1 (3.6%)	1 (11.1%)	0 (0%)	
Urinary tract infection	1 (3.6%)	1 (11.1%)	0 (0%)	
Ventilator-associated pneumonia	1 (3.6%)	1 (11.1%)	0 (0%)	
Outcomes				
Ventilator-day	10.0 (7.5–19.5)	10.0 (7.5–19.5)	NA	NA
Hospital-day	15.5 (13.0–22.8)	27.0 (17.5–34.0)	15.0 (11.0–19.0)	<0.01
Hospital-mortality	0 (0%)	0 (0%)	0 (0%)	>0.99

Data were presented as median (interquartile range) and frequency (percentage). Abbreviations: COVID, Coronavirus disease 2019; NMBA, neuromuscular blocking agent.

**Table 3:** Spearman correlation coefficients among absolute neutrophil counts, lymphocyte counts, and levels of biomarkers.

		ANC	ALC	CRP	Ferritin	LDH	D-Dimer
ANC	Spearman r	1.00	0.01	0.10	0.22	0.47	0.58
	Sig. (2-tailed)		0.91	0.40	0.20	<0.01	<0.01
	N	94	94	74	35	78	94
ALC	Spearman r	0.01	1.00	−0.42	−0.08	−0.20	−0.07
	Sig. (2-tailed)	0.91		<0.01	0.64	0.08	0.52
	N	94	95	74	35	79	95
CRP	Spearman r	0.10	−0.42	1.00	0.57	0.33	0.06
	Sig. (2-tailed)	0.40	<0.01		<0.01	0.01	0.57
	N	74	74	82	39	70	82
Ferritin	Spearman r	0.22	−0.08	0.57	1.00	0.49	0.38
	Sig. (2-tailed)	0.20	0.64	<0.01		<0.01	0.01
	N	35	35	39	42	39	42
LDH	Spearman r	0.47	−0.20	0.35	0.49	1.00	0.47
	Sig. (2-tailed)	<0.01	0.08	0.01	0.00		<0.01
	N	78	79	70	39	85	85
D Dimer	Spearman r	0.58	−0.07	0.06	0.38	0.47	1.00
	Sig. (2-tailed)	<0.01	0.52	0.57	0.01	<0.01	
	N	94	95	82	42	85	104

Abbreviations: ANC, absolute neutrophil counts; ALC, absolute lymphocyte counts; CRP, C-reactive protein; LDH, lactate dehydrogenase.

## Data Availability

The data in the present study are available upon request from the corresponding author.

## Supplementary Materials

The following are available online at https://www.mdpi.com/article/10.3390/jcm10173891/s1, Figure S1: Correlation between the level of D-Dimer and neutrophil count among patients with COVID categorized by distinct time periods.
