# Supplementary material for: Association between Early Absolute Neutrophil Count and Level of D-Dimer among Patients with COVID-19 Infection in Central Taiwan
Source: J Clin Med. 2021 Aug 30;10(17):3891. doi: 10.3390/jcm10173891 (PMC8432119; doi:10.3390/jcm10173891)
Supplement: Supplementary file 1 [file jcm-10-03891-s001.zip › jcm-1345348-supplementary.pdf]

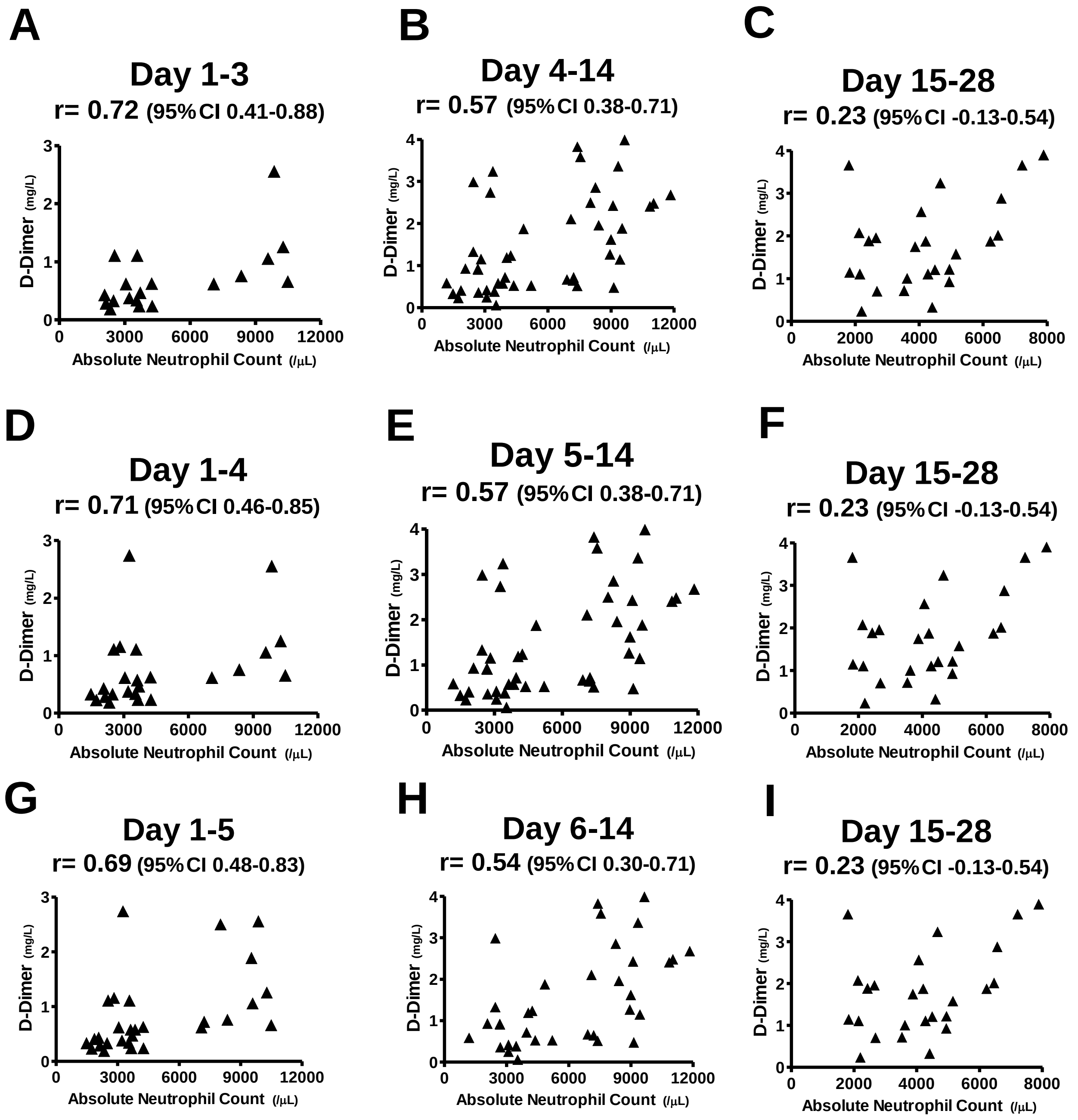

Supplemental Figure S1. Correlation between the level of D-Dimer and neutrophil count among patients with COVID categorised by distinct time periods
